# The emergence of plasmid-encoded oxacillinase and carbapenemase among uropathogenic *Escherichia coli* (UPEC) isolated from hospitalized patients in the North of Iran

**DOI:** 10.1016/j.heliyon.2023.e15386

**Published:** 2023-04-10

**Authors:** Mahsa Sadeghi, Ali Mojtahedi, Iraj Nikokar, Zahra Atrkar Roushan

**Affiliations:** a Burn and Regenerative Medicine Research Center, Guilan University of Medical Sciences, Rasht, Iran; b Department of Microbiology, School of Medicine, Guilan University of Medical Sciences, Rasht, Iran; c Medical Biotechnology Research Center, Faculty of Paramedicine, Guilan University of Medical Sciences, Rasht, Iran; d Department of Biostatistics, School of Medicine, Guilan University of Medical Sciences, Rasht, Iran

**Keywords:** Carbapenemase, *E. coli*, Oxacillinase, Urinary tract infection

## Abstract

Carbapenemase enzyme production is responsible for resistance to carbapenem among Gram-negative bacteria. This study aimed to detect common carbapenemase and oxacilinase genes among uropathogenic *E. coli* (UPEC) isolated from hospitalized patients in Rasht, north of Iran. In the present study, from 2000 urine samples, 263 UPEC strains were isolated from inpatients with urinary tract infections (UTI) in 2020. The Kirby-Bauer disk diffusion susceptibility test was used to determine the sensitivity or resistance of isolates to antimicrobial compounds. The double-disk test confirmed extended-spectrum β lactamase (ESBL) production phenotypically, and the presence and distribution of genes encoding carbapenemase and oxacilinase were investigated using polymerase chain reaction (PCR). Based on the findings, 13/263 isolates (8 ESBL and five non-ESBL) showed a non-susceptible phenotype to at least one of the studied carbapenem group antibiotics, and 121 (46%) isolates were ESBL-producers. PCR for oxacilinase and carbapenemase genes was done on all 126 isolates, including ESBL-positive and carbapenem-resistant strains, in which 10 (7.9%) and 25 (19.8%) isolates harbored *OXA-1* and *IMP* genes, respectively. Also, *OXA-2*, *OXA-10*, *OXA-48*, *VIM*, and *NDM* genes were not found in any studied isolates. *IMP* and *OXA-1* genes among carbapenemase-producing isolates indicate the possible spread of antibiotic-resistant strains. Hence, identification and control of ESBL and carbapenemase-producing strains, although with almost low frequency due to plasmid genes encoding carbapenemase, is essential for infection control.

## 1 Introduction

Drug-resistant Enterobacteriaceae, especially those producing ESBLs, are typically eradicated with carbapenems [1]. The worldwide emergence and spread of carbapenem-resistant Enterobacteriaceae (CRE) have become one of the most common problems in the healthcare system. There are a few options for treating CRE-infected patients because they are usually resistant to almost all beta-lactam antibiotics but susceptible to colistin and some aminoglycosides [2,3]. Limited and ineffective treatment options lead to increased morbidity and mortality. Determining these clinically important isolates' resistance mechanisms and geographic distribution is essential for infection control and public health measures [2]. The attributed mechanisms to *Enterobacteriaceae* for resistance to carbapenems include the following: (i) efflux pumps, (ii) acquisition of β-lactamases encoding genes, such as carbapenemase; and (iii) modification of porins in the outer membrane and penicillin-binding proteins [4]. According to Ambler's molecular classification, beta-lactamases are grouped into four distinct classes, A (ESBLs), B (MBLs), C (AmpC), and D (OXA-types), based on their amino acid sequence [5]. The class D β-lactamases (oxacillinases) are a global crisis and significant concern due to being encoded by transmissible genes. OXA β-lactamases are accountable for most resistance to β-lactams and revealed a high resistance to oxacillin and penicillin [5]. In recent years, the prevalence of isolates producing OXA enzymes with carbapenemase activity has increased significantly [6]. The isolates carry higher variants of OXA exhibit a high level of resistance to β-lactams, monobactams, and carbapenems as well as to aminoglycosides and fluoroquinolones [5]. Due to the increase of carbapenem-resistant *E. coli* in different regions of the world, periodic epidemiological surveillance and investigating the mechanisms of carbapenem-resistant isolates are essential for the global control of CRE [7]. The importance of the horizontal transfer of carbapenemase genes through mobile genetic elements and its relationship with increasing resistance to different groups of antibiotics has been highlighted [3]. Therefore, ongoing efforts are to develop early and more sensitive diagnostic methods for carbapenemase-producing isolates [4]. The detection of carbapenem-resistance genes leads to understanding the epidemiology and pathology of circulating resistant isolates and provides evidence for the design of appropriate antibiotic prescribing strategies [3]. Despite the increase in carbapenemase rate (especially MBL genes), data on antibiotic resistance and molecular epidemiology of carbapenem-resistant UPEC isolates are still limited. Therefore, this study was the second study conducted in recent years in northern Iran, aiming to determine the antibiotic resistance pattern and identify *bl-IMP*, *bl-NDM*, *bl-VIM*, and oxacillinase coding genes (*OXA-1*, *OXA-2*, *OXA-10*, *OXA-48*) among UPEC isolates.

## 2 Materials and methods

### 2.1 Study design, identification, and isolation of bacteria

In this cross-sectional study, 263 non-repetitive UPEC strains were isolated from 2000 urine samples related to inpatients who suffered from UTIs in 2020. The ethics of this study was confirmed by the Ethics Committee of Guilan University of Medical Sciences according to the declaration of Helsinki (IR.GUMS.REC.1397.230). All inpatients with suspected UTIs based on symptoms such as lower abdominal pain, frequency of micturition, hematuria, fever, burning micturition, painful micturition, nausea, or vomiting were included in this study. All inpatients who did not agree to participate in this study or had received antibiotics before sample collection were excluded from the study. Patients’ sterile urine samples were collected and cultured in Blood agar and EMB/MacConkey agar media (Merck, Darmstadt, Germany) separately and incubated (at 37 °C) for 24 h. Detection of ≥10^3^ CFU/ml from a neat sample for children and ≥10^5^ CFU/ml for adults was considered positive culture. Then, Biochemical tests and amplification of the specific region of 16srRNA (919 bp, F-AGAGTTTGATCMTGGCTCAG, R-CCGTCAATTCATTTGAGTTT) were used to identify UPEC strains [8].

### 2.2 Antibiotic susceptibility tests

All isolates were evaluated for antibiotic susceptibility pattern by the Kirby Bauer method on Mueller-Hinton agar medium (Merck, Germany) and interpreted according to Clinical and Laboratory Standards Institute (CLSI) protocol [9]. In this study, 18 antibiotic disks including Gentamicin (10 μg), Amikacin (30 μg), Meropenem (10 μg), Imipenem (10 μg), Ampicillin (10 μg), Aztreonam (30 μg), Tetracycline (30 μg), Ciprofloxacin (5 μg), Levofloxacin (5 μg), Norfloxacin (10 μg), Nalidixic acid (30 μg), Co-trimoxazole (25 μg), Nitrofurantoin (300 μg), Co-amoxiclav (30 μg), Cefepime (30 μg), Cefoxitin (30 μg), Ceftazidime (30 μg), and Ceftriaxone (30 μg) (MAST, U.K.) were tested. The plates were inspected after aerobic incubation at 37 °C for 16–20 h. *UPEC* ATCC 25922 was used for quality control purposes.

### 2.3 Test for ESBL producing isolates

Based on CLSI recommendation, a double-disk synergy test was used to determine ESBLs production using ceftazidime (30 μg) and cefotaxime (30 μg) in combination with clavulanic acid (10 μg) disks [9]. UPEC ATCC 25922 and *Klebsiella pneumoniae* ATCC 700603 were applied for negative and positive control, respectively. After incubation for 24 h at 37 °C, an increase of ≥5 mm in the inhibition zone diameter of the combined disk compared to a single disk was represented as ESBL production [10].

### 2.4 DNA extraction and polymerase chain reaction

Per the manufacturer's instructions, pure overnight cultures were subjected to genomic DNA extraction using an extraction kit (Roche, Germany). The oxacilinase [*OXA-1*, *OXA-2*, *OXA-10*, and *OXA-48*] and carbapenemase [*VIM*, *NDM*, and *IMP*] genes were individually targeted to amplify on a thermal cycler Simpliamp (Applied Biosystems, USA) using the seven specific primers (Metabion Co, Germany) shown in Table 1. The PCR conditions for the amplification of the studied genes were as follows: one cycle of 5 min at 95 °C for pre-incubation and 30 cycles of denaturing at 95 °C for 30 s, followed by annealing for 30 s (annealing temperature is highly dependent on the melting temperature of primers), extension at 72 °C for 60 s and a final extension at 72 °C for 5 min. Enterobacter isolates carrying carbapenemase and oxacillinase genes, confirmed in our colleague's research in Shiraz, was used as the positive control [11,12]. The amplicons were separated on 1.5% agarose gel and visualized by ultraviolet light (Cinnagen Co., Iran).

**Table 1:** Primers sequences used for amplification of target genes in this study.

Genes	primers	Sequence 5ˈ→3ˈ	Product size (bp)	Ref.
*OXA-1*	Forward	AGCCGTTAAAATTAAGCCC	909	[13]
	Reverse	CTTGATTGAAGGGTTGGGCG		
*OXA-2*	Forward	AAGAAACGCTACTCGCCTGC	485	[14]
	Reverse	CCACTCAACCCATCCTACCC		
*OXA-10*	Forward	TCAACAAATCGCCAGAGAAG	276	[15]
	Reverse	TCCCACACCAGAAAAACCAG		
*OXA-48*	Forward	GCGTGGTTAAGGATGAACAC	438	[16]
	Reverse	CATCAAGTTCAACCCAACCG		
*VIM*	Forward	GATGGTGTTTGGTCGCATA	390	[17]
	Reverse	CGAATGCGCAGCACCAG		
*IMP*	Forward	ACCGCAGCAGAGTCTTTGCC	232	[17]
	Reverse	ACAACCAGTTTTGCCTTACC		
*NDM*	Forward	GGTTTGGCGATCTGGTTTTC	621	[18]
	Reverse	CGGAATGGCTCATCACGATC		

### 2.5 Statistical methods

Statistical analysis was performed using the IBM SPSS software (version 21.0, IBM, USA). Chi-square or Fisher's exact tests were applied for data analysis. Also, descriptive statistics were used to present the results regarding relative frequency. A p-value less than 0.05 was set as statistically significant.

## 3 Results

The antibiotic susceptibility of 263 UPEC isolates was evaluated against 18 antimicrobials. Of which, the highest susceptibility to meropenem (99.2%) and imipenem (95.8%) and the lowest susceptibility against ampicillin (16.3%) and nalidixic acid (22.8%) were observed. In addition, carbapenems were the most effective agents against ESBL isolates in laboratory conditions. Among all UPEC isolates, 13 (4.94%) cases were non-susceptible (intermediate/resistant) to at least one of the studied carbapenem group antibiotics. Of these, 7 (54%) and 6 (46%) strains were isolated from women and men, respectively. 11 (4.2%) and 2 (0.8%) isolates were non-susceptible (intermediate/resistant) to imipenem and meropenem, respectively. Phenotypic findings showed among 263 tested isolates, 121 (46%) strains harbored ESBL, and 142 (54%) cases were negative. PCR for oxacilinase and carbapenemase genes was done on all 126 isolates, including 121 ESBL positive and five carbapenem-resistant strains; the amplification of *OXA-1* and *IMP* genes revealed 10 (7.9%) and 25 (19.8%) isolates harbored these genes, respectively. Also, *OXA-2*, *OXA-10*, *OXA-48*, *VIM*, and *NDM* genes were not found in any studied isolates. All strains harboring *IMP* and *OXA-1* genes were ESBL positive, while no significant relationship between the existence of these genes and ESBL phenotype was found. The distribution of genes encoding carbapenemase and oxacilinase concerning antibiotic resistance and ESBL phenotypes are shown in Table 2. Based on our findings, there was no significant association between resistance to imipenem and meropenem and the presence of carbapenem-resistance genes (P = 0.62 and P = 0.64). Also, no significant relationship was detected between carbapenem-resistance genes and UPEC with/without ESBL. Finally, a significant association was observed between the presence of the *OXA-1* gene and resistance to imipenem (P < 0.05).

**Table 2:** Distribution of genes in relation with carbapenem resistance and ESBL phenotype.

Genes						
Antibiotic Pattern	Imipenem		Meropenem		ESBL	
	Resistant^a^ No. (%)	Susceptible No. (%)	Resistant No. (%)	Susceptible No. (%)	Negative No. (%)	Positive No. (%)
*IMP*-positive	2 (8)	23 (92)	0	25 (100)	0	25 (100)
*IMP*-negative	9 (8.9)	92 (91.1)	2 (2)	99 (98)	5 (5)	96 (95)
P-value	0.62		0.64		0.32	
*VIM*-positive No. (%)	0	0	0	0	0	0
*VIM*-negative No. (%)	11 (8.7)	115 (91.3)	2 (1.6)	124 (98.4)	5 (4)	121 (96)
P-value	ND		ND		ND	
*NDM*-positive No. (%)	0	0	0	0	0	0
*NDM*-negative No. (%)	11 (8.7)	115 (91.3)	2 (1.6)	124 (98.4)	5 (4)	121 (96)
P-value	ND		ND		ND	
*OXA-1*-positive No. (%)	3 (30)	7 (70)	1 (10)	9 (90)	0	10 (100)
*OXA-1*-negative No. (%)	8 (6.9)	108 (93.1)	1 (0.9)	115 (99.1)	5 (4.3)	111 (95.7)
P-value	0.04		0.15		0.65	
*OXA*-*2*-positive No. (%)	0	0	0	0	0	0
*OXA*-*2*-negative No. (%)	11 (8.7)	115 (91.3)	2 (1.6)	124 (98.4)	5 (4)	121 (96)
P-value	ND		ND		ND	
*OXA*-*10*-positive No. (%)	0	0	0	0	0	0
*OXA*-*10*-negative No. (%)	11 (8.7)	115 (91.3)	2 (1.6)	124 (98.4)	5 (4)	121 (96)
P-value	ND		ND		ND	
*OXA*-*48*-positive No. (%)	0	0	0	0	0	0
*OXA-48*-negative No. (%)	11 (8.7)	115 (91.3)	2 (1.6)	124 (98.4)	5 (4)	121 (96)
P-value	ND		ND		ND	

a Include intermediate-resistant isolates.

## 4 Discussion

The emergence and spread of carbapenemase-producing bacteria is an emergent and challenging global problem because most of these isolates are multidrug-resistance [19]. Therefore, surveillance and early identification of these strains are essential. Since phenotypic methods usually have less sensitivity and specificity than genotypic methods, molecular techniques are sometimes used [20]. Accurate identification of carbapenemase-producing isolates is essential to understand the prevalence and implement contact precautions. In the present study, the most effective antibiotics were imipenem and meropenem; their resistance rates were reported as 4.2% and 0.8%, respectively. The prevalence of the *IMP* gene was 19.8%, while *VIM* and *NDM* genes were not found in any of the samples. According to our results, there was no significant relationship between resistance to imipenem and meropenem and the presence of carbapenemase-coding genes. Resistance in these strains can be caused by other genes or mechanisms of resistance to carbapenems, such as resistance mediated by purines and efflux pumps [21]. One of the most important mechanisms of resistance to carbapenem is the production of class D beta-lactamase (OXA). In our study, a significant association was observed between the presence of the *OXA-1* gene and resistance to imipenem. Similar to our study, studies conducted in Iran indicate the high susceptibility of *UPEC* isolates to carbapenem antibiotics (imipenem and meropenem); For instance, the study of Abdollahi (86.9%), Shahcheraghi (96.3%), and Moayednia (98.8%) [22,23]. Also, in the studies by Enwuru in Nigeria and Yayan in Germany, all *UPEC* isolates were susceptible to imipenem [24,25]. In the study of Nojoomi in 2017, among 50 carbapenem-resistant *UPEC* isolates, the prevalence of the *IMP* gene was 16%, and no *VIM* and *NDM-1* genes were reported, which was very similar to our results [1]. Contrary to our results, in the Peirano study, 38%, 2%, and 1% of isolates contained the *NDM*, *VIM-1*, and *IMP* genes, respectively [26]. Also, a study in southeast Iran in 2015 showed that none of the ESBL-positive *E. coli* isolates harbored *VIM* and *IMP* genes. In contrast, a study in the north of Iran in 2020 reported the frequency of *blaIMP*, *blaVIM*, and *blaOXA-48* genes among UPEC isolates as 8.7%, 9.8%, and 15.3%, respectively [1,20]. In our study, the frequency of the *OXA-1* gene was 7.9%, and no *OXA-2*, *OXA-10*, and *OXA-48* genes were found. In a survey by Chaudhary in India, out of 49 phenotypically oxacillinase-producing *UPEC* isolates, 16 isolates (32.6%) had the *OXA-48* gene, eight isolates (16.3%) had *OXA-10*, and five isolates (10.2%) included *OXA-1*, *OXA-2*, *OXA-51*, and *OXA-58*, which did not match to our results [5]. Also, in the study of Alizadeh in the southeast of Iran (Kerman), the rate of *OXA-1* was 17.2%, which was higher than our study [27]. The prevalence of the *OXA-48* gene in the study of Nojoomi was 8%, and in other studies, it was 11% in hospitalized patients and 6% in outpatients [1,28]. Even though the prevalence of *OXA-2*, *OXA-10*, and *OXA-48* genes were low in these studies, they did not match our results. In general, a variable rate of prevalence of carbapenem-resistant *E. coli* has been reported worldwide. These differences can be due to the overuse or incorrect antibiotic prescribing, self-curing, improper infection control policies in medical centers, and inexact diagnostic methods for detecting resistant isolates. Although carbapenemase and oxacillinase genes were rarely reported in this review, strains containing these genes are still accountable for resistance to some antibiotics. Therefore, these strains detection, tracking, and control of these strains are necessary due to the plasmid genes encoding carbapenemase and easy transfer among other bacteria. Periodic surveillance to know the dispersal and mechanisms of antibiotic resistance plays an essential role in preventing and overcoming the risk of complications caused by ESBL and carbapenemase-producing strains. In addition, monitoring antibiotic resistance patterns can effectively set healthcare guidelines and rational antibiotic prescriptions.

## 5 Conclusion

Carbapenems were still the most effective antibiotics against strains, especially ESBL producers. This study showed the existence of MBL and oxacillinase-producing strains in the common urinary tract infection pathogen. The emergence and spread of this type of strain should be considered an alarming risk because these drug-resistant isolates can spread quickly. Therefore, early identification of infections related to carbapenem resistance genes and prevention of its dissemination by screening, hygiene measures, and isolation of carriers is necessary. Besides that, using advanced diagnostic facilities and the rational use of antibiotics can reduce the incidence and outbreak of resistant isolates.

### 5.1 Limitations

This research included some limitations. First, the sample size was relatively small, and only samples obtained from one hospital were examined. Second, we could not assess the presence of other OXA-type carbapenemase genes to more accurately evaluate oxacillinase resistance genes among the isolates. Third, the presence of a gene does not always mean gene expression, so methods such as real-time PCR should be used to confirm gene expression. Due to financial constraints, we could not use this technique.

## Author contribution statement

Mahsa Sadeghi: Performed the experiments; Wrote the paper.

Ali Mojtahedi: Conceived and designed the experiments.

Iraj Nikokar: Contributed reagents, materials, analysis tools or data.

Zahra Atrkar Roushan: Analyzed and interpreted the data.

## Data availability statement

Data included in article/supp. material/referenced in article.

## Declaration of interest’s statement

The authors declare no conflict of interest.

## Additional information

No additional information is available for this paper.

## Funding support statement

This work was financially supported by Guilan University of Medical Sciences, Rasht, Iran (Grant number: 97052012).
